# A Normal FGF23 Does Not Preclude Tumor‐Induced Osteomalacia

**DOI:** 10.1002/jbm4.10438

**Published:** 2020-12-23

**Authors:** Neeharika Nandam, Sadia Ejaz, William Ahrens, Maya Styner

**Affiliations:** ^1^ Department of Medicine, Division of Endocrinology and Metabolism University of North Carolina at Chapel Hill Chapel Hill NC USA; ^2^ Department of Pathology Carolinas Medical Center Charlotte NC USA

**Keywords:** DISORDERS OF CALCIUM/PHOSPHATE METABOLISM, TUMOR‐INDUCED BONE DISEASE, PTH/Vit D/FGF23, OSTEOMALACIA AND RICKETS, ORTHOPEDIC INJURY/FRACTURE HEALING

## Abstract

Tumor‐induced osteomalacia (TIO) is a rare cause of impaired bone mineralization mediated by the osteocyte‐derived, phosphaturic hormone: fibroblast growth factor 23 (FGF23). The case is presented of a previously healthy 45‐year‐old man who developed fragility fractures at multiple sites (initially metatarsals, eventually ribs, hips, spine, scapula, and sacrum) resulting in rapid functional deterioration, weakness, and the inability to bear weight and ambulate without a walker. Workup for secondary causes of bone loss was negative except for mild hypogonadotropic hypogonadism with normal pituitary MRI and hypophosphatemia that persisted despite aggressive supplementation. Testosterone was initiated but discontinued 6 months later because of deep vein thrombosis and pulmonary embolism, likely provoked by his new sedentary state, in addition to smoking history and possibly testosterone usage. Serum FGF23 was nonelevated at 138 mRU/mL (44–215). A genetic panel for OI variants was negative for a causal mutation. At the age of 48, 3 years after his initial fracture, he was referred to our academic endocrine clinic. We ruled out additional mutations that lead to hypophosphatemic rickets, including phosphate‐regulating endopeptidase homolog, X‐linked. PET/CT looking for a potential TIO locus revealed uptake in the left suprapatellar recess. Biopsy was consistent with a phosphaturic mesenchymal tumor. FGF23 was repeated for a preoperative baseline and now found to be elevated at 289 mRU/mL. In retrospect, it is likely that the initial level was inappropriately elevated for the degree of hypophosphatemia. After resection, he experienced marked improvement in physical function, decreased pain, and resolution of renal phosphate wasting. The principals of establishing a robust clinical diagnosis of TIO should be emphasized, excluding other entities and avoiding pitfalls in the interpretation of laboratory testing. © 2020 The Authors. *JBMR Plus* published by Wiley Periodicals LLC. on behalf of American Society for Bone and Mineral Research.

## Case Presentation

A 45‐year‐old previously healthy white man presented with multiple fragility fractures over 2 years, as well as pain and functional deficits eventually rendering him nonambulatory. He carried no previously diagnosed medical history, although he was a former smoker with a 13 pack‐year smoking history. His family history was notable for grade 1 chondrosarcoma in his mother. He initially presented to an orthopedic clinic with foot pain and hip pain after starting an exercise program. Initially, he was counseled to participate in physical therapy; however, he continued to develop progressively disabling pain. Over the course of 8 months, he was diagnosed with bilateral metatarsal and subtrochanteric femur fractures on imaging.

A local endocrinologist was consulted based on suspicion for secondary causes of bone loss. His laboratory tests were notable for a low phosphorus at 2.1 mg/dL (2.7–4.5), elevated alkaline phosphatase (ALP) at 155 IU/L (34–104), elevated PTH at 211 pg/mL (12.0–72.0), low 25OHD, and 1,25(OH)_2_D_3_ at 27 ng/mL (30–80) and 12 pg/mL (20–80), respectively. However, calcium was normal at 8.8 mg/dL (8.4–10.4). Serum protein electrophoresis revealed no evidence of monoclonal gammopathy. Additional studies included a low free testosterone at 4.4 ng/mL (5–21) and luteinizing hormone at 2.2 mIU/mL (3.0–10.0). Other pituitary hormone levels and brain MRI were unremarkable.

**Fig 1:**
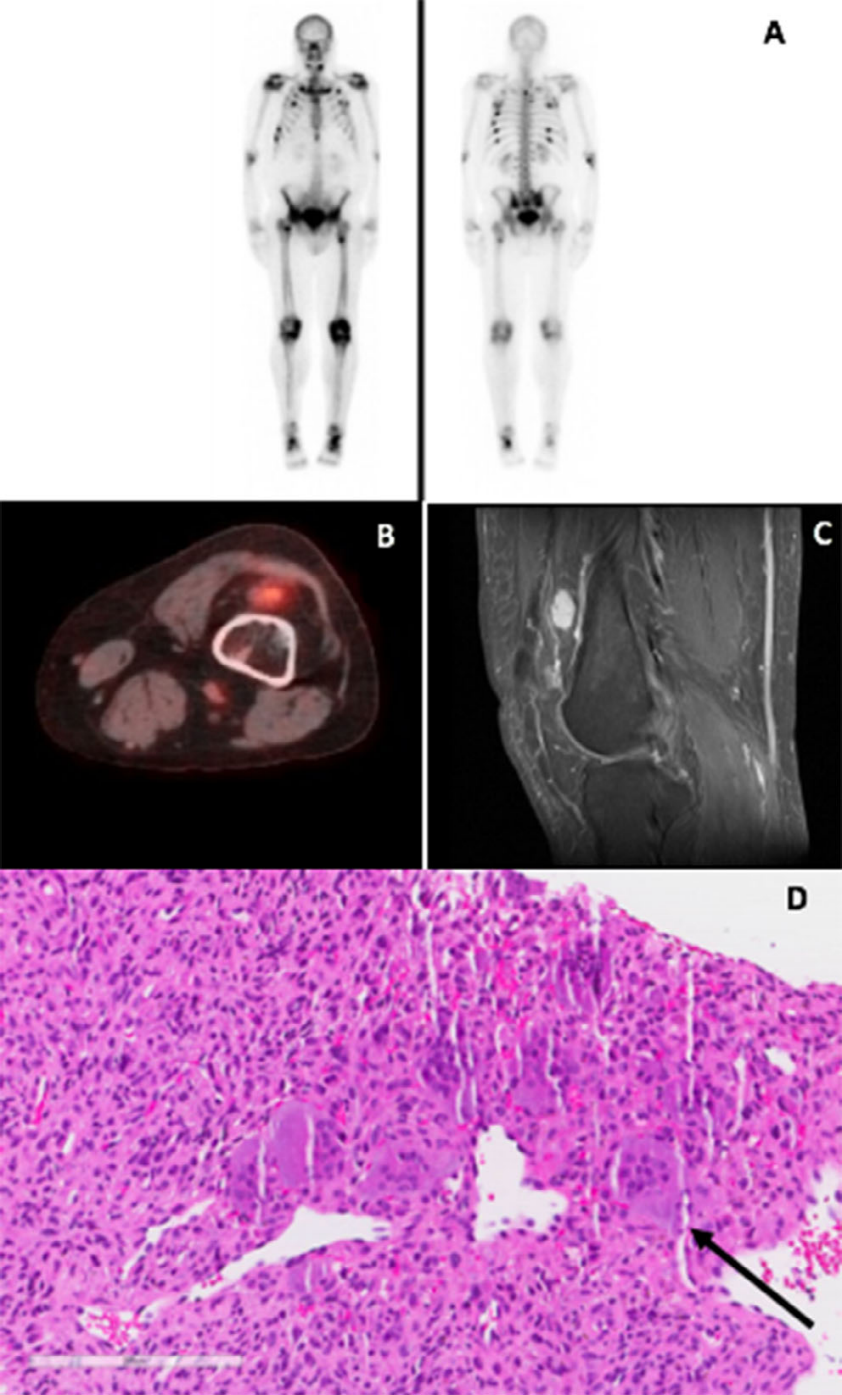
Key radiologic imaging and histology sections from our patient with tumor‐induced osteomalacia (TIO). Top panel: (*A*) 99mtechnetium methylene diphosphonate (99mTc MDP) bone scan shows increased uptake in bilateral anterior/posterior ribs, bilateral sternoclavicular joints, right proximal intertrochanteric femur, inferior sternum, multiple bilateral lumbar transverse processes, right posterior scapula, posterior elements along upper thoracic spine, posterior elements along lumbosacral junction, right posterior sacrum, bilateral ankles, likely bilateral mid‐feet. Overall impression: Extensive multifocal regions with increased uptake likely representing subacute, remodeling, or other active stress fractures. Middle panel: (*B*) Computed tomography/fluorodeoxyglucose ‐positron emission tomography (CT/FDG PET) scan shows an area of suprapatellar enhancement that was suspicious for a TIO locus (*C*) Sagittal MR image showing the patient's hypermetabolic lesion measuring 1.7 cm in largest dimension in the left suprapatellar recess, and corresponding to hypermetabolic area on PET/CT. Bottom panel: (*D*) suprapatellar tumor surgical pathology specimen stained with H&E: Pattern is consistent with phosphaturic mesenchymal tumor. Arrow indicates multinucleated giant cells.

Vitamin D3, calcitriol, phosphate, and testosterone were prescribed. Testosterone was discontinued 6 months later, after developing a venous thromboembolism of the left common femoral, superficial femoral, and popliteal veins, and pulmonary embolus of the left pulmonary artery. Workup for genetic causes of hypercoagulability was negative; he ultimately received anticoagulation for a year with rivaroxaban. Incidental rib fractures on a chest X‐ray at the time prompted a three‐phase ^99^Tc‐MDP bone scan, revealing multiple sites of uptake: ribs, scapulae, sternum, thoracolumbar spine, sacrum, bilateral ankles, and feet *(*Fig. 1*A)*.

Laboratory tests showed ongoing phosphate wasting, despite compliance with calcitriol 0.25 μg/d, cholecalciferol 5000 IU/d, and phosphorus 2250 mg/d in divided doses. Calculated tubular reabsorption of phosphate was 64% (>80%) and renal tubular maximal phosphate to glomerular reabsorption rate (TmP/GFR) was 1.74 mg/dL (2.5–4.5), indicating inappropriately low Pi reabsorption. Of note, it is unclear if the patient was taking phosphorus supplementation at the time of these measurements. Urine 24‐hour calcium was 244.8 mg (100–300). Testing for causal mutations in hypophosphatemic rickets and osteogenesis imperfecta was negative. Serum C‐terminal FGF23 level was tested based on concern for an acquired cause of hypophosphatemia such as TIO, and was within the reference range at 138 RU/mL (LabCorp ELISA 44–215; LabCorp, Burlington, NC, USA). At this point, 3 years after symptom onset, he was referred to our academic endocrinology practice for further workup of possible secondary causes of his phosphate wasting. His examination was remarkable only for an elevated BMI of 35, diffuse muscle and bone tenderness to palpation without evident deformities, and requirement for an ambulation‐assist device. We interpreted his normal serum FGF23 level as inappropriately high for his degree of hypophosphatemia, prompting a search for a suspected TIO locus, which was further considered based on rapid clinical deterioration in a previously healthy patient. 18F‐fluorodeoxyglucose positron emission tomography (^18^F‐FDG‐PET) exhibited a hypermetabolic focus at the left suprapatellar recess, which corresponded with a small 1.7‐cm mass that had previously been seen on CT 2 years prior but thought to be clinically insignificant because of its benign appearance (Fig. 1*B*). MRI demonstrated stability in size over this time period with near homogenous enhancement suggestive of a benign process; signal intensity was slightly higher than muscle with punctate areas showing low signal foci, potentially indicative of a degree of mineralization (Fig. 1*C*). Serum C‐terminal FGF23 measurement was measured with LabCorp ELISA again, and was now elevated at 289 RU/mL (44–215).

Biopsy showed a phosphaturic mesenchymal tumor (Fig. 1*D*) and chromogenic in situ hybridization (Mayo Clinic, Rochester, MN, USA) was positive for FGF23 mRNA, confirming this site as a likely TIO locus (Table 1). After surgical resection of the tumor, serum C‐terminal FGF23 declined to <50 RU/mL 1‐week postoperative and was 90 RU/mL 3 weeks later (Mayo <180 RU/mL). Phosphorus and 25OHD level were rechecked at 1 month postsurgery and found to have normalized, permitting discontinuation of calcitriol and phosphorus. DEXA 8 weeks after surgery showed marked improvements, with lumbar spine *T* score increasing from −2.8 to −1.0 (BMD +64.29%) and femoral neck *T* score from −1.9 to −1.4 (BMD +26.32%). The patient also experienced significant improvement in physical function and pain after his operation.

**Table 1:** Clinical Characteristics Prior to and After Tumor Resection

Test	Years prior to presentation				Weeks after surgery				Range
	3	1	0.5	Initial visit	1	4	12	32	
Phosphorus serum (mg/dL)		2.0	2.4			3.9	4.1	2.8	2.5–5.0
Urine spot ‐ Phos (mg/dL) ‐ Cr (mg/dL)			Phos: 57 Cr: 47						Urine Phos: N/A Urine Cr: 30–125
TmP/GFR (mg/dL)			1.74						2.4–4.5
24‐h urine calcium (mg/24 h)		244.8		90.0					<100 Low Ca diet <300 Normal diet
Alk phos (IU/L)		166		177	99	126	125	125	34–104
iPTH (pg/mL)		105		211	69.2			62	12.0–72.0
Calcium (mg/dL)		9.2		8.7		9.2	9.3	9.3	8.6–10.3
25OHD (ng/mL)				24		33	41	31	30–80
1,25(OH)_2_D (pg/mL)				12.0				55.6	19.9–79.3
FGF23 (mRU/mL)					<50	90			Mayo ELISA <180
			138	289					LabCorp ELISA 44–215
Pituitary hormones	LH (mIU/mL): 2.2 ACTH (pg/mL): 24.5 TSH (mIU/mL): 2.6 Prolactin (ng/mL): 4.6		LH 1.6 FSH (mIU/mL): 1.7						LH: 1.2–8.6 FSH: 1.3–19.3 ACTH: 7.2–63.3 TSH: 0.3–4.5 Prolactin: 4.0–15.2
24‐h urine–free cortisol (μg/dL/24 h)				12.0					0–50.0
Testosterone Total (ng/dL) Free (pg/mL)	Total 175 Free 4.4		Total 151 Free 6.6						Total: 249–836 Free: 6.8–21.5
SPEP		No monoclonal gammopathy							N/A
Genetic testing		OI panel negative	CTGT panel negative						
Imaging studies	**MRI L Foot**: Transverse fracture second MT, marrow edema, also medial cuneiform **Hip X‐rays:** Stress fractures‐ RFN & Left subtrochanteric **CT and MRI lower extr:** fractures as above, and with benign appearing L suprapatellar soft tissue nodule	**3‐phase** ^**99**^ **Tc‐MDP bone scan:** Multiple stress fractures **MRI Brain:** normal pituitary		**DEXA** Spine T − 2.8 FN T − 1.9 **PET‐CT**: hypermetabolic L suprapatellar lesion **MRI L knee**: stable appearing L suprapatellar lesion		**MRI L Knee:** no evidence of soft tissue lesion recurrence		**DEXA** Spine T − 1.0 FN T − 1.4	

Connective tissue gene tests abnormal mineralization disorder panel, next‐generation sequencing. All coding regions for genes on the panel were analyzed for variants using Illumina (San Diego, CA, USA) MiSeq next‐generation sequencers (ALPL, ANKH, CASR, CLCN5, CYP27B1, DMP1, ENPP1, FAH, FGF23, OCRL, PHEX, SLC34A1, SLC34A3, SLC9A3R1, VDR).

Abbreviations: 25OHD = calcidiol; 1,25(OH)_2_D = calcitriol; ACTH = adrenocorticotropic hormone; Alk phos = alkaline phosphatase; CT = computed tomography; CTGT = connective tissue gene test; DEXA = dual energy X‐ray absorptiometry; ELISA = enzyme‐linked immunosorbent assay; FSH = follicle‐stimulating hormone; iPTH = intact parathyroid hormone; LH = luteinizing hormone; MRI = magnetic resonance imaging; MT = metatarsal; OI panel = osteogenesis imperfecta panel from Invitae analyzed clinically important regions of each specified gene (*COL1A1, COL1A2, CRTAP, P3H1*); PRL = prolactin; SPEP = serum protein electrophoresis; TMP/GFR = tubular max reabsorption of phosphate.

## Background

In the late 1950s the Swiss pediatric endocrinologist Andrea Prader was the first to identify a case of acquired hypophosphatemia caused by a ricketogenic substance [FGF23].^(^ ^1^, ^2^, ^3^ ^)^ It would take more than 40 years to clone this humoral factor, or phosphatonin, causing renal phosphate wasting.^(^ ^4^ ^)^ Studies of autosomal dominant hypophosphatemic rickets led to identifying FGF23 as the most common phosphatonin, crucial in both physiologic phosphate regulation and the driver of phosphate wasting in multiple diseases.^(^ ^4^, ^5^, ^6^ ^)^

Bone‐derived FGF23 is upregulated with increased serum inorganic phosphate (Pi) and downregulated in the setting of hypophosphatemia.^(^ ^7^ ^)^ Interestingly, tight regulation of FGF23 degradation, rather than its synthesis, permits its secretion.^(^ ^8^ ^)^ GALNT3 functions to O‐glycosylate FGF23, thus protecting FGF23 from degradation and permitting its release.^(^ ^9^ ^)^ On the other hand, the absence of the zinc metallopeptidase phosphate‐regulating endopeptidase homolog, X‐linked (PHEX) lowers FGF23 via unclear mechanisms.^(^ ^10^ ^)^ Intact FGF23, secreted by osteocytes and their precursors, binds with coreceptor α‐klotho to FGFR1 in the renal proximal tubule, reducing expression of the sodium‐phosphate cotransporters NaPi‐2a and NaPi‐2c, and ultimately increasing urinary phosphate excretion.^(^ ^11^ ^)^ In addition to its effect on renal Pi handling, FGF23 suppresses renal 1‐α‐hydroxylase (CYP27B1), thereby lowering calcitriol synthesis; FGF23 also upregulates vitamin D 24‐hydroxylase (CYP24), inactivating calcitriol.^(^ ^9^, ^11^ ^)^ In addition to FGF23 and vitamin D, PTH is also an important phosphate regulator. PTH (via PTHR1) promotes phosphaturia by a mechanism similar to FGF23, however in contrast, upregulates CYP27B1 and suppresses CYP24.^(^ ^12^ ^)^

The presence of multiple fragility fractures in young adults merits consideration of secondary causes of bone loss. The differential diagnosis includes disorders of the collagen matrix such as osteogenesis imperfecta and disorders of calcium and vitamin D metabolism.^(^ ^13^ ^)^ Hypophosphatemia suggests a genetic versus acquired cause of Pi loss.^(^ ^14^ ^)^ Common acquired causes of low Pi include primary hyperparathyroidism, secondary hyperparathyroidism from vitamin D deficiency, and alcohol abuse.^(^ ^12^, ^14^ ^)^ Fanconi syndrome presents with glycosuria and aminoaciduria in addition to phosphaturia; it can be either inherited or secondary to medications or other illnesses, such as multiple myeloma.^(^ ^12^, ^15^ ^)^ Distinguishing whether hypophosphatemia is driven by renal phosphate wasting is a key part of diagnostic evaluation, and is done by calculating the tubular maximal reabsorption rate of phosphate to glomerular filtration (TmP/GFR).^(^ ^16^ ^)^ Causes such as hyperparathyroidism and Fanconi syndrome with renally mediated Pi losses typically have low TmP/GFR values, whereas extrarenal causes such as excess phosphate binder intake and refeeding syndrome should have appropriately high TmP/GFR for the degree of hypophosphatemia.^(^ ^16^ ^)^

If investigations reveal renal‐mediated hypophosphatemia unexplained by the above causes, serum FGF23 is measured. High FGF23, or a level inappropriately normal for the degree of hypophosphatemia, might indicate a disorder of FGF23 excess. X‐linked hypophosphatemic rickets (*PHEX*) and autosomal dominant hypophosphatemic rickets (*FGF23*) are genetic disorders resulting in decreased FGF23 breakdown.^(^ ^12^ ^)^ Of note, several patients with *FGF23* mutations, especially women, may be normophosphatemic during childhood; thus testing for this mutation should be part of the workup for adult onset fragility fractures with hypophosphatemia.^(^ ^12^ ^)^ Autosomal recessive hypophosphatemic rickets type 1 (*DMP1*) leads to increased transcription of FGF23.^(^ ^9^, ^12^ ^)^ McCune‐Albright syndrome (*GNAS* somatic mutation) may rarely cause FGF23 overexpression.^(^ ^12^ ^)^ Additionally, ferric carboxymaltose administration can cause osteomalacia through an increase in FGF23.^(^ ^17^ ^)^

TIO is caused by overproduction of FGF23 by small, typically benign mesenchymal tumors, leading to fragility fractures and diffuse bone and muscle pain.^(^ ^18^ ^)^ Laboratory findings are similar to those of the FGF23 excess syndromes described above, with hypophosphatemia, renal phosphate wasting, and low to inappropriately normal 1,25(OH)_2_D_3_ for the degree of hypophosphatemia, however, without a previous history of these lab abnormalities.^(^ ^19^ ^)^ Serum calcium, 25OHD, and PTH levels are normal, though persistently low 1,25(OH)_2_D_3_ may lead to secondary hyperparathyroidism, with elevated ALP.^(^ ^19^ ^)^

The time from symptom onset to diagnosis is over 2.5 years in most cases of TIO, in part because of delays in initial testing for hypophosphatemia, but also because of difficulty in localizing tumors.^(^ ^19^ ^)^ Functional imaging, which takes advantage of the high expression of somatostatin receptors in TIO, is recommended and uses ^111^In‐octreotide or ^68^Gallium tetraazacyclododecanetetraacetic acid–DPhe1‐Tyr3‐octreotate (^68^Ga‐DOTATATE) for tumor localization.^(^ ^11^ ^)^ DOTATATE PET/CT likely has the greatest sensitivity and specificity for TIO, with octreotide scanning also being a sensitive imaging method based on the presence of somatostatin receptors in TIO.^(^ ^11^, ^20^ ^)^ However, ^18^FDG‐PET‐CT is useful if somatostatin‐based scans are negative.^(^ ^11^, ^20^ ^)^ In some cases, biopsy has been discouraged based on the possibility of tumor seeding, with venous sampling being an alternate option if further diagnostic clarification is needed.^(^ ^14^ ^)^ Successful tumor resection typically results in skeletal healing and reversal of biochemical defects; excision with wide, tumor‐free margins is essential because of the risk of tumor recurrence.^(^ ^14^, ^19^ ^)^ Histopathology shows a mesenchymal tumor of mixed connective tissue variant (PMT‐CT).^(^ ^21^ ^)^ Immunohistochemical staining or RT‐PCR‐based detection of FGF23 mRNA transcription is often used to demonstrate increased FGF23 expression.^(^ ^21^, ^22^ ^)^ In our patientʼs case, chromogenic in situ hybridization was used to support the diagnosis.

## Discussion

This case serves as an example of the importance of considering TIO in the differential diagnosis of fragility fractures, particularly with the constellation of new‐onset persistent renal phosphate wasting in the absence of genetic causes of hypophosphatemic osteomalacia. The presence of FGF23 level in the normal range should be interpreted as inappropriately elevated and potentially suggestive of TIO, as physiologically FGF23 should be downregulated with hypophosphatemia.

Interestingly, our patient's serum FGF23 level was elevated only on recheck 5 months after his initial level. Although his tumor could have expressed higher amounts of FGF23 mRNA during this period, this case also brings into question the reproducibility of FGF23 level and sensitivity of commercially available serum assays. For example, in a study by Imel and colleagues, the test sensitivity of one C‐terminal FGF23 assay (Immutopics, Inc., San Clemente, CA, USA) was 73% in TIO; although this specific assay is different from that used for our patient, this suggests that C‐terminal assays might miss a fraction of TIO cases.^(^ ^23^ ^)^ Imel and colleague's study also investigated the an intact FGF23 (iFGF23; Kainos Laboratories, Tokyo, Japan) assay and Immutopics intact assay (now discontinued), which had sensitivities of 86% and 23%, respectively.^(^ ^23^ ^)^ Of note, Mayo Clinic Laboratories has recently started offering an iFGF23 assay.^(^ ^24^ ^)^ Further research examining the sensitivities of currently available assays, including head‐to‐head comparisons of C‐terminal to iFGF23 assays, will be important for improving diagnostic accuracy in TIO. Alternatively, lowering the cutoff used for TIO diagnosis may help improve test sensitivity. For example, Proposals have been made for using iFGF23 values just above the population median as a threshold for ruling in TIO, rather than the upper limit of normal; it is possible that a similar principle may apply to C‐terminal assays.^(^ ^25^ ^)^

Alternate testing modalities are needed for cases not easily diagnosed via imaging and/or FGF23 measurement. Venous sampling measuring FGF23 may have clinical utility in verifying a suspicious mass on imaging as being a TIO locus, and systematic sampling may guide locations for further imaging in patients with unrevealing radiographic studies.^(^ ^26^, ^27^ ^)^ Of note, current studies of venous sampling have been conducted using iFGF23 measurements, with subjects usually having elevated levels.^(^ ^26^, ^27^ ^)^ It is difficult to say if any of these patients would have had a normal C‐terminal FGF23, similar to our patient, if checked. Whether venous sampling may be of clinical utility in patients with normal iFGF23 or C‐terminal FGF23 levels merits further study, as this may be a relevant method in cases such as ours.

In some TIO patients with normal FGF23 levels, one may consider hypophosphatemia driven by a different paraneoplastic phosphaturic hormone, or whether FGF23 secretion may be partially responsive to serum phosphate levels in some tumors.^(^ ^23^ ^)^ For example, matrix extracellular phosphoglycoprotein, secreted frizzled protein 4, and FGF‐7 are all additional phosphatonins that have rarely been associated with TIO.^(^ ^12^, ^28^ ^)^

Of note, this patient's family history of chondrosarcoma raises the question of a genetic predisposition to developing TIO tumors in patients with a family history of skeletal malignancy. For example, an *FN1‐FGFR1* fusion gene has been identified in several TIO tumors; this gene has been hypothesized to cause tumorigenesis in TIO through FGF23 binding, leading to autocrine or paracrine activation of the receptor tyrosine kinase.^(^ ^14^, ^29^ ^)^ Interestingly, *FGFR1* fusion genes have been identified as pathogenic in the 8p11 myeloproliferative syndrome, breast cancer, glioblastoma, and lung squamous cell carcinoma.^(^ ^29^, ^30^ ^)^ Additionally, similar microRNA profiles were recently noted in osteosarcomas and TIO; both show upregulation of the biomarker miR‐197 and downregulation of miR‐20b, miR‐144, and miR‐335.^(^ ^31^ ^)^ Further genetic studies of TIO may improve our understanding of the disease and identify patients missed by currently available modalities.

## Conclusion

This case illustrates a potential pitfall in the diagnosis of tumor‐induced osteomalacia (TIO), highlighting that a normal serum C‐terminal or intact FGF23 might not exclude the disorder in a patient with high clinical suspicion based on acquired hypophosphatemic osteomalacia. Rather, a normal C‐terminal or intact FGF23 must be interpreted as inappropriately high in the setting of hypophosphatemia and warrants a search for FGF23‐excess syndromes such as TIO.

## Disclosures

All authors report that there are no relevant conflicts of interest, no relevant financial or nonfinancial relationships, no patents (whether planned, pending, or issued) broadly relevant to this work, or any other relationships/conditions/circumstances that present a potential conflict of interest.

## AUTHOR CONTRIBUTIONS

**Neeharika Nandam:** Conceptualization; data curation; investigation; project administration; writing‐original draft; writing‐review and editing. **Sadia Ejaz:** Conceptualization; writing‐original draft; writing‐review and editing. **William Ahrens:** Data curation; resources; software; visualization; writing‐review and editing. **Maya Styner:** Conceptualization; data curation; investigation; project administration; writing‐original draft; writing‐review and editing.
